# Prognostic Value of Cerebrovascular Reactivity (PRx) Versus Intracranial Pressure (ICP) Monitoring in Traumatic Brain Injury: Systematic Review

**DOI:** 10.3390/jcm15124611

**Published:** 2026-06-14

**Authors:** Bartosz Rodziewicz, Mikołaj Kacperski, Justyna Małgorzata Fercho, Oskar G. Chasles, Jacek Szypenbejl, Mariusz Siemiński

**Affiliations:** 1 Scientific Circle of Neurotraumatology, Department of Emergency Medicine, Medical University of Gdansk, 80-210 Gdansk, Poland; m.kacperski@gumed.edu.pl (M.K.); oskar.chasles@gumed.edu.pl (O.G.C.); jacek.szypenbejl@gumed.edu.pl (J.S.); 2 Department of Emergency Medicine, Medical University of Gdansk, 80-210 Gdansk, Poland; mariusz.sieminski@gumed.edu.pl; 3 Neurosurgery Department, 10th Military Research Hospital and PolyClinic SPZOZ, 85-681 Bydgoszcz, Poland; 4 Lung Transplant Unit, Cardiac Surgery Department, Medical University of Gdansk, 80-210 Gdansk, Poland

**Keywords:** traumatic brain injury, intracranial pressure, pressure reactivity index, cerebrovascular reactivity, cerebral autoregulation, prognosis, mortality

## Abstract

**Background**: Intracranial pressure (ICP) monitoring remains the cornerstone of neurocritical care in severe traumatic brain injury (TBI), yet its prognostic value as a standalone metric is limited. The Pressure Reactivity Index (PRx), a continuous measure of cerebrovascular reactivity derived from ICP and arterial blood pressure, may offer additional or complementary prognostic information. This systematic review aimed to compare the prognostic performance of PRx-derived metrics versus standard ICP monitoring for mortality and functional outcome in patients with TBI. **Methods**: A systematic search of PubMed, Web of Science, and Scopus was conducted for studies published between January 2000 and December 2025. Studies were eligible if they included adult TBI patients with continuous multimodal monitoring and reported comparative prognostic data for PRx- and ICP-based metrics. Risk of bias within the studies was appraised via the QUIPS tool, and the GRADE system was used to rate the strength of the evidence. Due to methodological heterogeneity, findings were synthesized narratively. **Results**: Nine studies were included. Applying a maximum-cohort estimation to account for overlapping registries, the pooled sample comprised a minimum of 1240 unique patients. In the majority of included studies, direct within-cohort head-to-head comparisons demonstrated that specific PRx-derived metrics—such as the individualized ICP threshold (iICP), Longest Continuous Duration of Autoregulatory Impairment (LCAI), Lower Limit of Reactivity (LLR), and time-integrated burdens (%Time > Threshold)—yielded stronger prognostic discrimination compared to standard ICP thresholds for both mortality (PRx: AUC 0.747–0.648 and ICP: AUC 0.660–0.614) and functional outcome. When added to established predictive models, PRx-derived metrics provided clinically meaningful incremental improvements in prognostic accuracy, with descriptive incremental AUC gains ranging from +0.039 to +0.170 across the six studies reporting model augmentation. Due to heterogeneity in baseline models, PRx-derived metrics, and patient populations, these findings are presented strictly as a descriptive range. **Conclusions**: PRx and PRx-derived cerebrovascular reactivity metrics-namely iICP, LCAI, LLR, and time-integrated burdens of autoregulatory failure—show potential to offer additive prognostic value beyond standard ICP monitoring in severe TBI. However, because current evidence is strictly observational and likely influenced by institutional confounders, it cannot currently support definitive clinical recommendations. Further prospective, multicenter studies utilizing standardized thresholds are necessary to confirm these associative findings and isolate their true prognostic value.

## 1. Introduction

Traumatic brain injury (TBI) remains a global health threat, impacting roughly 69 million individuals every year and standing as a primary driver of death and long-term disability [1]. For those with severe injuries, the prognosis is particularly poor: mortality rates range from 30% to 40% [2], while the annual economic impact is estimated to surpass $400 billion [3]. This burden is exacerbated by the fact that TBI disproportionately impacts younger populations [4]. Despite decades of progress in neurocritical care, finding ways to reliably improve long-term outcomes remains a persistent clinical challenge [3].

Clinical management of severe TBI has been anchored to intracranial pressure (ICP) control for nearly five decades [5]. Ever since Lundberg’s early contributions [6], the prevailing logic has been simple: keep ICP below the 20–22 mmHg mark to prevent secondary injury [7]. The limitation of this uniform approach, however, is that it does not account for the dynamic nature of cerebral autoregulation, which fluctuates substantially across different patients [8]. A single static ICP reading may therefore be misleading, as it can represent entirely different underlying cerebrovascular risk profiles despite identical absolute values [9,10]. This inherent lack of nuance is likely why ICP, when used in isolation, remains a somewhat blunt tool for prognosis, as evidenced by a modest AUC of 0.69 for mortality prediction [11].

The Pressure Reactivity Index (PRx) offers a more dynamic alternative. By calculating a moving correlation between slow waves of ICP and mean arterial pressure, PRx provides a continuous, real-time window into cerebrovascular reactivity [12]. Negative or near-zero values suggest that the brain is successfully autoregulating, whereas positive values point toward a dangerous, pressure-passive state [13]. More importantly, by allowing for estimation of an individualized “optimal” cerebral perfusion pressure (CPPopt), PRx signals a transition from static pressure targets toward physiology-led, bespoke management [14,15].

Despite its physiological appeal, PRx has yet to be firmly integrated into international guidelines. Its true prognostic edge over standard ICP monitoring is still a point of contention [16], largely because the existing body of research is fragmented by varied study designs and inconsistent outcome definitions. To our knowledge, no systematic review has yet performed a direct head-to-head comparison of PRx- and ICP-based metrics in matched cohorts [17,18], nor has any study clearly determined whether PRx adds meaningful data to established models based on admission characteristics [2].

Consequently, this systematic review aims to objectively synthesize the available evidence comparing the predictive performance of PRx- and ICP-based metrics. We focused specifically on evaluating their comparative discrimination (AUC), statistical independence, and incremental value when assessed within the same patient cohorts. To expand upon prior literature, this review provides a clearer separation of standalone versus augmented AUCs and shows a strict prognostic-versus-therapeutic distinction. Clarifying the strictly prognostic utility of PRx is a necessary step to determine its potential role in individualized TBI management strategies.

## 2. Materials and Methods

### 2.1. Study Design and Registration

This systematic review was conducted and reported in accordance with the Preferred Reporting Items for Systematic Reviews and Meta-Analyses (PRISMA) guidelines [19] (see File S1 for detailed checklist). The review protocol was prospectively registered in the International Prospective Register of Systematic Reviews (PROSPERO) under the registration number CRD420251266228.

### 2.2. Search Strategy and Data Sources

A systematic review was undertaken to find research comparing the predictive validity of the Pressure Reactivity Index (PRx) against traditional Intracranial Pressure (ICP) measurements following traumatic brain injury. The search was executed across three major electronic databases: PubMed, Web of Science (WoS), and Scopus. The search terms consisted of both free-text words and Medical Subject Headings (MeSH), which were queried across the title and abstract fields. The search strings encompassed four key conceptual domains: (1) target population (“Traumatic Brain Injury”, “severe head injury”), (2) primary physiological metric (“Pressure Reactivity Index”, “PRx”, “cerebral autoregulation”), (3) core comparator (“intracranial pressure”, “ICP”), and (4) clinical outcomes (“mortality”, “prognosis”). The exact search strings utilized for each database are provided in the Supplementary Material (File S2). To restrict the results to contemporary and highly relevant evidence, the search was limited to human studies published in English between 1 January 2000 and 7 December 2025. Studies published online ahead of print were eligible if the final publication year was 2025. Publication types such as reviews, meta-analyses, editorials, case reports, meeting abstracts, and book chapters were systematically excluded using database-specific filters. Deduplication was performed using a custom Python (Python Software Foundation, Wilmington, DE, USA) script based on matching of DOIs and article titles.

### 2.3. Study Selection and Eligibility Criteria

Following deduplication, a two-stage screening process (title/abstract screening followed by full-text review) was performed independently by two investigators (B.R. and M.K.). The inter-rater agreement between the two independent reviewers was evaluated using Cohen’s kappa, yielding a coefficient of κ = 0.80 for the title and abstract screening phase and κ = 0.71 for the full-text screening phase. Any discrepancies or conflicts regarding study eligibility were resolved by more experienced researchers (M.S. and J.S.). Studies were included if they met the following predefined criteria:

Population and Context: Adult patients (≥16 years old) with acute TBI requiring admission to an Intensive Care Unit (ICU). The clinical setting included either specialized Neurocritical Care Units or general ICUs capable of invasive multimodal monitoring. There were no geographical restrictions. Patients must have undergone continuous invasive multimodal monitoring, specifically including Intracranial Pressure (ICP) and Arterial Blood Pressure (ABP), to enable PRx calculation.

Intervention (Exposure): Studies reporting on prognostic metrics derived from continuous cerebrovascular reactivity monitoring. The primary parameter required was the Pressure Reactivity Index (PRx), which utilizes continuous ICP and Mean Arterial Pressure (MAP) data. The exposure had to be explicitly related to clinical outcomes.

Comparator: Comparison of the prognostic performance of PRx-derived metrics versus conventional ICP-based metrics, reflecting the design of the included studies in which both monitoring parameters were assessed within the same patients rather than a comparison of distinct treatment strategies.

Outcomes: Reporting of standard clinical endpoints, specifically mortality or functional neurological outcome (Glasgow Outcome Scale [GOS] or Extended GOS [GOSE]).

Study Design: Both randomized and nonrandomized study designs were eligible for inclusion.

Studies focusing on pediatric populations (<16 years old), animal models, non-traumatic brain injuries, or those lacking continuous multimodal monitoring to enable PRx calculation were excluded.

### 2.4. Data Extraction

Data extraction was performed by two reviewers (B.R. and M.K.), with any disagreements resolved by a third investigator (J.F.), who made the final decision when necessary. The extracted variables included:

General study information: Primary author, year published, research design, along with the underlying cohort or data source.

Patient demographics: Sample size (N), median age, and baseline Glasgow Coma Scale (GCS) score.

Monitoring variables: Evaluated physiological metrics, including mean ICP, PRx, and derived parameters: individualized ICP threshold (iICP), %Time > Threshold, Lower Limit of Reactivity (LLR), and Longest Continuous duration of Autoregulatory Impairment (LCAI).

Statistical findings: Primary predictive measures for clinical outcomes, focusing on the Area Under the Receiver Operating Characteristic Curve (AUC), Odds Ratios (OR), and the incremental value of adding PRx-derived metrics to baseline clinical models.

### 2.5. Quality Assessment

The methodological quality and potential risk of bias of the included prognostic studies were evaluated using the Quality in Prognosis Studies (QUIPS) tool [20]. Risk of bias was appraised across six specific categories: study participation and attrition, the measurement of both prognostic factors and outcomes, study confounding, along with statistical analysis and reporting. The overall risk of bias for each study was subsequently categorized based on these domains.

### 2.6. Data Synthesis

Because of considerable expected heterogeneity in study methods—especially concerning PRx thresholds, prognostic modeling, and the reporting of endpoints—conducting a meta-analysis was not viable. Therefore, the evidence was synthesized narratively. The extracted data were grouped and analyzed according to the primary outcomes: mortality and functional outcome. The comparative predictive performance (AUC, OR) and the incremental prognostic value (ΔR^2^) of PRx-derived metrics versus standard ICP were systematically summarized to identify overarching trends in the evidence base. To rigorously account for overlapping clinical material across major registries (Cambridge, CENTER-TBI, and Canadian databases), a conservative maximum-cohort estimation was applied. Specifically, to mitigate overcounting, the total pooled patient count was calculated by aggregating only the single largest sample size from each distinct database, rather than summing the cohorts of all included studies. Because patient overlap was not directly verified with the original authors, we explicitly flag the assumption of nested cohorts, making this total a structural estimate rather than a confirmed count. To evaluate the robustness of the synthesized findings, a sensitivity analysis was performed by restricting the narrative synthesis exclusively to studies appraised as having a low overall risk of bias via the QUIPS tool. Data visualization, specifically the generation of Figures, was performed using Python version 3.12 [21].

### 2.7. Certainty of Evidence

The overall confidence in the data regarding principal outcomes was rated using the Grading of Recommendations Assessment, Development and Evaluation (GRADE) tool [22].

### 2.8. AI Statement

During the preparation of this manuscript, the authors used Gemini 3.1 Pro (Google LLC, Mountain View, CA, USA) for stylistic editing. The authors have reviewed the output and take full responsibility for the content of this publication.

## 3. Results

### 3.1. Study Selection

**Figure 1:**
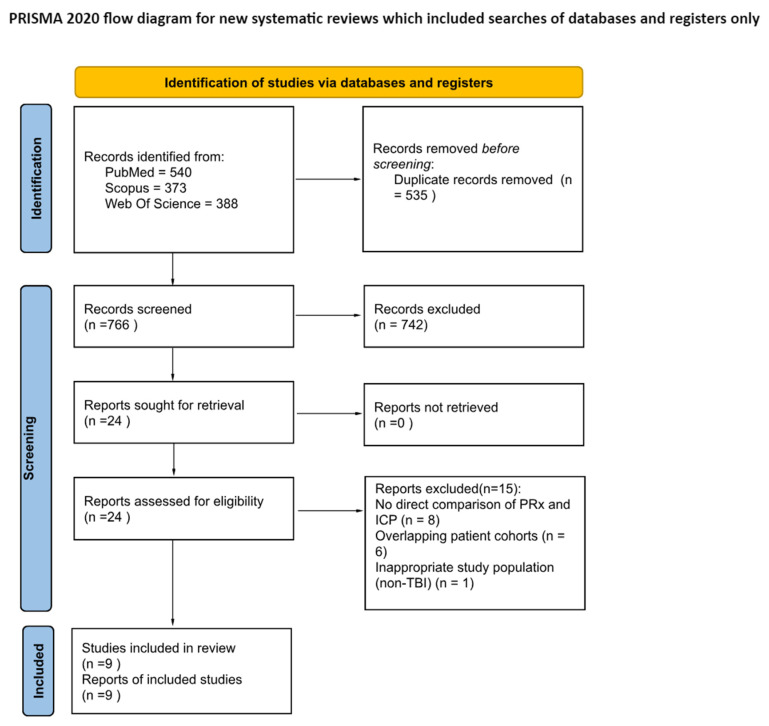
PRISMA Flow Diagram.

The database search retrieved 1301 records. After removal of 535 duplicates, 766 unique records underwent screening; 742 were excluded at title/abstract stage. Of 24 reports retrieved for full-text review, 15 were excluded: no direct PRx–ICP comparison (*n* = 8), potential cohort overlap (*n* = 6), non-TBI population (*n* = 1). The six studies excluded on grounds of cohort overlap included those drawing from the same source databases (notably CENTER-TBI), and their exclusion was intended to reduce the risk of double-counting patients in the final synthesis. Despite excluding six studies for apparent overlap, multiple included reports still drew from shared datasets. Applying our predefined maximum-cohort estimation, we report a conservative pooled minimum of 1240 unique patients. Nine studies were included in the final narrative synthesis (Figure 1).

### 3.2. Characteristics of Included Studies

The nine included studies encompassed estimated 1240 unique patients with acute TBI, published between 2007 and 2025. Seven were retrospective observational; two were prospective. Cohorts originated from Cambridge, UK (*n* = 3), the pan-European CENTER-TBI registry (*n* = 3), Canada (*n* = 2), and Lithuania (*n* = 1). Median patient age ranged from 34 to 53 years; admission GCS ranged from 5 to 7. Sample sizes ranged from 43 to 601. Detailed information is presented in Table 1.

### 3.3. Summary of Risk of Bias Assessment

**Figure 2:**
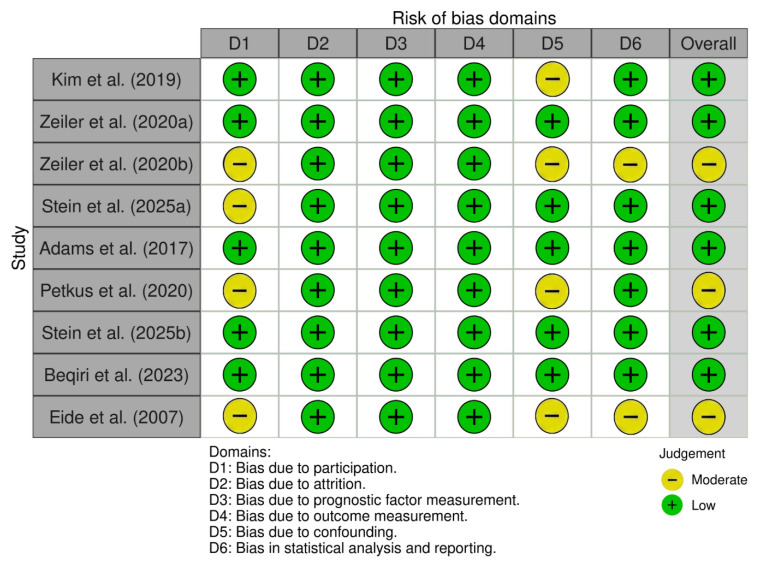
Risk of Bias Assessment. Derived from [23,24,25,26,27,28,29,30,31].

Six of nine studies (67%) were classified as low overall risk of bias [23,24,26,27,29,30]. Three studies received a moderate rating [25,28,31]. No study was rated high risk. The most frequent concern was D5 (confounding), flagged in four studies due to limited covariate adjustment beyond ICP and PRx. Domains D2–D4 were consistently low across all studies (Figure 2).

### 3.4. Summary of Evidence Certainty 

Assessment using the GRADE framework [22] resulted in a low to very low level of certainty, reflecting the observational nature of the included studies. The evidence for both mortality prediction and incremental prognostic value was rated as LOW (⊕⊕◯◯). This rating was due to serious inconsistency across the literature, driven by conflicting PRx thresholds, baseline models, and parameters. For functional outcomes (GOS/GOSE), the evidence was rated as VERY LOW (⊕◯◯◯) due to a contradiction in the data: retrospective studies showed a clear link to recovery, whereas the sole prospective cohort found no statistically significant benefit. The complete GRADE summary of findings is provided in the Supplementary Material (File S3).

### 3.5. Predictive Performance for Mortality

Seven studies evaluated mortality, directly comparing the prognostic discrimination of PRx-derived metrics against standard static ICP thresholds. Kim et al. [23] observed that the duration of PRx > 0.20 yielded higher discrimination for mortality than mean ICP alone (AUC 0.77 vs. 0.66, *p* < 0.001). Zeiler et al. [25] reported that PRx > 0.25 yielded a higher AUC as a univariate predictor of death (0.747, *p* = 0.002) than the conventional ICP > 20 mmHg threshold (0.648, *p* = 0.006). The utility of dynamic targets was further noted by Stein et al. [26], who found that individualized ICP (iICP) provided higher mortality prediction than standard ICP (AUC 0.660, *p* = 0.029 vs. 0.614, *p* = 0.021) and established a significant discrimination threshold for survival (χ^2^ = 5.48, *p* < 0.05). Beqiri et al. [30] observed that elevated ICP (>20 mmHg) was significantly associated with mortality primarily when it coincided with cerebral perfusion pressure (CPP) dropping below the PRx-derived lower limit of reactivity (LLR) (AUC 0.73, *p* < 0.001), suggesting a prominent role for autoregulatory hypoperfusion in patient outcomes. Adams et al. [27] showed that a PRx-based model yielded higher discrimination for early fatal outcomes (0–48 h) relative to an ICP-based model (AUC 0.86 vs. 0.80, *p* < 0.05). Finally, Petkus et al. [28] identified the longest continuous duration of autoregulatory impairment (LCAI) as a significant independent predictor of mortality (OR 29.62; 95% CI 2.09–419.07, *p* = 0.012), whereas the addition of standard ICP parameters did not further improve the model’s overall prognostic accuracy.

### 3.6. Predictive Performance for Functional Outcome

Four studies evaluated functional outcomes (GOS/GOSE), comparing the prognostic utility of PRx-derived metrics with standard ICP parameters. Eide et al. [31] reported that PRx values differed significantly between patients with unfavorable and favorable outcomes (0.20 vs. 0.03, *p* < 0.01), acting as an independent predictor in multivariate analysis alongside mean ICP (PRx: *p* = 0.03; mean ICP: *p* = 0.04). Zeiler et al. [25] found PRx > 0.25 to be a significant univariate predictor of GOSE (AUC 0.679, *p* = 0.034), whereas standard ICP > 20 mmHg did not reach statistical significance (*p* > 0.05). Stein et al. [29] observed that iICP demonstrated a higher correlation with autoregulatory failure burden (r = 0.49) compared to the standard ICP > 20 mmHg threshold (r = 0.14). Conversely, a prospective analysis by Zeiler et al. [24] indicated that incorporating PRx-derived metrics into baseline predictive models did not yield a statistically significant improvement in discrimination for GOSE over models utilizing ICP alone (*p* > 0.05).

### 3.7. Incremental Prognostic Value of PRx-Derived Metrics Added to Predictive Models

**Figure 3:**
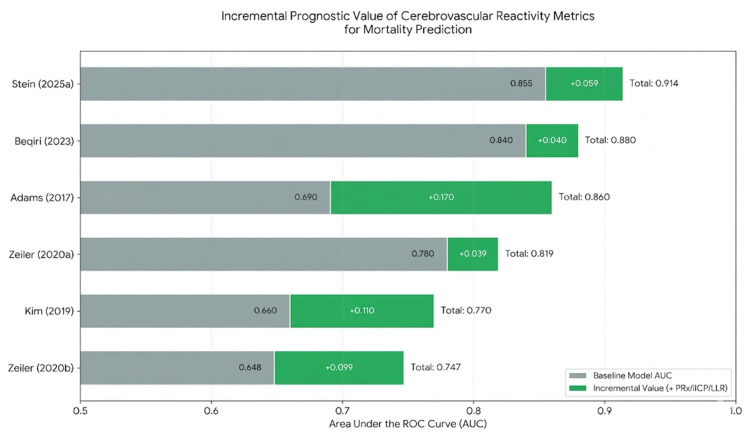
Incremental AUC Values. Derived from [23,24,25,26,27,30].

Six studies formally assessed the incremental prognostic value of PRx-derived metrics when added to baseline predictive models incorporating standard ICP (Figure 3). Stein et al. [26] reported that adding iICP to the IMPACT Core model increased the AUC to 0.914, yielding an additional variance (ΔR^2^ = 0.263) compared to standard ICP limits (ΔR^2^ = 0.156). Beqiri et al. [30] observed that incorporating the PRx-based LLR into the IMPACT Core model increased both the AUC (0.84 to 0.88) and adjusted R^2^ (0.49 to 0.57, *p* < 0.001), yielding higher predictive metrics than models based solely on ICP. Adams et al. [27] indicated that incorporating PRx into a static clinical model increased early-phase prediction accuracy (AUC 0.69 to 0.86, *p* < 0.05), with PRx maintaining independent predictive status alongside ICP (OR 11.43, *p* = 0.001). Zeiler et al. [24] reported that adding the percentage of time with PRx > 0.25 to an ICP-inclusive baseline model increased the AUC from 0.780 to 0.819 (*p* < 0.0001; ΔPseudo-R^2^ = 10.4%). In patients with intracranial hypertension, Kim et al. [23] showed that the duration of PRx > 0.20 yielded an absolute AUC increase of +0.11 over mean ICP alone. Finally, Petkus et al. [28] reported that a multi-factorial model based on the PRx-derived LCAI metric achieved 88.6% accuracy, with the addition of standard ICP/CPP parameters providing no further incremental prognostic value. Across the six studies reporting a quantifiable AUC gain, the incremental improvement ranged from +0.039 to +0.170. These studies utilized markedly different baseline models, PRx-derived metrics, and cohort sizes. Specifically, more modest incremental AUC gains were observed when adding the percentage of time with PRx > 0.25 (+0.039), LLR (+0.040), or iICP (+0.059) to baseline IMPACT Core models. Larger predictive differences emerged when evaluating PRx > 0.25 directly against standard ICP > 20 mmHg limits (+0.099), comparing cumulative PRx > 0.20 duration versus mean ICP in patients with intracranial hypertension (+0.110), or adding continuous PRx to a static clinical model to predict early fatal outcomes (+0.170). Therefore, due to this substantial methodological heterogeneity, pooling these findings into a single average is misleading, and they are presented strictly as a descriptive range.

### 3.8. Sensitivity Analysis

To assess the stability of the synthesized evidence, a sensitivity analysis was performed by excluding the three studies categorized as having a moderate risk of bias (Zeiler et al., 2020b; Petkus et al., 2020; Eide et al., 2007) [25,28,31]. Consequently, specific findings derived from these cohorts warrant cautious interpretation. These include the identification of LCAI as an independent mortality predictor with a high odds ratio (Petkus et al. [28]), the maximum standalone predictive discrimination of PRx > 0.25 (AUC 0.747; Zeiler et al., 2020b [25]), and the multivariate significance of continuous PRx for functional recovery (Eide et al. [31]).

However, restricting the overall analysis to the remaining six studies with a low risk of bias [23,24,26,27,29,30] confirmed the stability of the model augmentation findings. Following the exclusion of moderate-RoB data, the descriptive range of incremental AUC improvements for mortality prediction models—the primary measure of additive value in this review—remained entirely unaffected (+0.039 to +0.170).

## 4. Discussion

### 4.1. Pathophysiological Rationale: The Shift from Static ICP to Dynamic Autoregulation

The findings of this review must be interpreted within the broader pathophysiological context of cerebral autoregulation in TBI. The current standard of care—targeting ICP below 20–22 mmHg—is founded on a population-derived threshold rather than an individualized physiological target [7]. While this approach has underpinned neurocritical care guidelines for decades [5], it fails to account for the substantial inter-individual variability in cerebrovascular responses following TBI [8]. A patient may maintain ICP within the accepted range while simultaneously exhibiting profound failure of cerebrovascular autoregulation—a state that static pressure monitoring cannot detect [9].

Cerebral autoregulation—the intrinsic capacity of the cerebrovascular system to maintain stable perfusion across a range of arterial pressures—is frequently disrupted following TBI [32,33]. PRx captures this disruption continuously, providing a real-time index of reactivity that static ICP cannot [12]. A key insight emerging from the studies included in this review is the concept of cumulative autoregulatory burden: it is not a single transient ICP elevation, but the sustained duration of impaired reactivity that drives secondary neuronal injury. This is most clearly illustrated in this review by Kim et al. [23] and Petkus et al. [28], whose data demonstrate that time-integrated metrics of autoregulatory failure—cumulative PRx > 0.20 duration and LCAI, respectively—outperformed mean ICP as predictors of mortality. These findings align with experimental evidence that prolonged pressure-passive cerebral states lead to progressive ischemic injury, uncoupled from absolute ICP levels [34,35].

### 4.2. Additive Value of PRx-Derived Metrics in Prognostication

The prognostic data synthesized in this review suggest a potential additive value of PRx-derived metrics over standard ICP monitoring. For mortality prediction, direct within-study comparisons demonstrated that PRx yielded higher diagnostic discrimination than static ICP thresholds when evaluated head-to-head in the exact same patient cohorts. When evaluated strictly as standalone predictors, AUC values for PRx-based parameters ranged from 0.64 to 0.75, compared to 0.61 to 0.65 for standalone static ICP thresholds—a pattern observed across geographically and methodologically diverse cohorts. These findings support prior observations suggesting that dynamic cerebrovascular indices capture prognostic variance that pressure monitoring alone cannot explain [36,37].

Notable is the evidence for incremental model augmentation. When PRx-derived parameters were formally added to established prognostic frameworks—most notably the IMPACT Core model—consistent and statistically significant improvements in predictive accuracy were observed across all six studies reporting this outcome, with incremental AUC gains ranging from +0.039 to +0.170. Because these studies varied significantly in their baseline clinical models, specific PRx parameters tested, and cohort sizes, this finding is presented strictly descriptively as a range. In the context of prognostic medicine, such an increment is considered clinically meaningful; for comparison, the addition of CT characteristics to the IMPACT Core model yields improvements of a comparable magnitude [38]. The findings of Stein et al. (2025a) [26] are particularly striking, with iICP increasing IMPACT Core AUC from 0.793 to 0.914—a gain that substantially exceeds what standard ICP parameters could provide in the same model (AUC 0.855).

The temporal analysis by Adams et al. [27] further reveals that the prognostic advantage of PRx is strongly time-windowed [39]. The authors demonstrated that the predictive power of a PRx-based model for fatal outcomes is highest during the early acute phase (0–48 h; AUC 0.86) but significantly decays over time (AUC dropping to 0.74 at 240 h). This time-dependency carries profound implications for bedside management and resource allocation. It suggests that continuous cerebrovascular reactivity monitoring is most critical during the initial 48-h window, where early autoregulatory failure drives the most severe secondary injury propagation [40]. Consequently, these findings suggest that the clinical utility of PRx is maximized when early autoregulatory data is prioritized to identify patients at the highest risk of acute secondary injury. Not all findings were uniformly positive. Zeiler et al. (2020a) [24] reported that while PRx-derived metric significantly improved mortality prediction, improvements in functional outcome (GOSE) discrimination did not reach statistical significance. This discrepancy likely reflects the multifactorial nature of long-term functional recovery, which extends beyond cerebrovascular physiology to encompass rehabilitation access, pre-injury status, and neuroplasticity [41,42]. It also highlights the need for future prognostic models that integrate physiological monitoring data with broader clinical and social variables.

### 4.3. Clinical Implications: Moving Toward Personalized Neurocritical Care

The aggregate evidence presented in this review highlights the theoretical potential of PRx in clinical practice, but a strict distinction must be maintained between prognostic prediction and therapeutic efficacy. Concepts such as CPPopt or iICP represent a physiological shift from population-level thresholds toward patient-specific profiling [14,17]. Continuous PRx monitoring allows clinicians to identify a patient’s unique autoregulatory window, and metrics like iICP demonstrate a stronger correlation with autoregulatory failure burden than static 20 mmHg limits [29]. However, it is crucial to emphasize that the studies included in this review only demonstrate that PRx-derived metrics predict clinical outcomes. No included study evaluated whether actively titrating hemodynamic and intracranial interventions to these dynamic PRx-guided targets actually improves patient survival or functional recovery.

Although PRx has not yet been incorporated into major international TBI management guidelines [7,43], its promising prognostic performance supports the case for further evaluation within multimodal neuromonitoring protocols. For instance, the observation by Beqiri et al. [30] that high ICP was most lethal when coincident with CPP falling below the PRx-derived LLR suggests that reactivity-informed monitoring can identify high-risk physiological states that standard ICP limits might miss [44]. Nevertheless, until randomized controlled trials prospectively demonstrate that managing patients strictly according to these dynamic targets leads to superior clinical outcomes compared to standard care, PRx must be regarded primarily as an advanced prognostic monitoring tool rather than a validated therapeutic intervention. Furthermore, any future translation of these metrics into routine bedside practice will necessitate consensus-based efforts to standardize monitoring durations and validate specific PRx thresholds across neurocritical care settings [45,46].

### 4.4. Limitations

This review has several limitations, mainly due to the observational design of the included studies.

First, the methods for calculating and applying PRx varied widely. Studies used different definitions for pathological reactivity, ranging from fixed traditional thresholds (PRx > 0.20 or >0.25) to dynamically optimized cutoffs (PRx > +0.05). This heterogeneity stems from both methodological approaches and inherent patient characteristics. For instance, patient age significantly alters physiological tolerance; as demonstrated by Petkus et al., younger patients (<45 years) tolerated a higher failure burden (mean PRx > 0.36), whereas older patients (>45 years) experienced mortality at much lower thresholds (mean PRx > 0.26). Conversely, methodological evolution has shifted focus toward dynamically optimized, patient-specific targets rather than fixed population limits. This lack of consensus prevents a formal meta-analysis and makes it difficult to establish standardized bedside protocols.

Second, the findings may not be widely generalizable due to geographic and institutional biases. The cohorts came almost exclusively from specialized academic neurotrauma centers in Europe and Canada, so the performance of PRx in non-specialist or lower-resource hospitals remains unknown. Additionally, centers equipped for high-resolution cerebrovascular monitoring often differ from non-monitoring centers in their overall TBI management, staffing levels, and access to specialists. These institutional differences may independently improve patient outcomes, acting as a confounder when assessing the prognostic value of PRx.

Third, the existing literature rarely stratifies patients by injury mechanism (blunt vs. penetrating TBI) or systemic burden (isolated brain injury vs. severe polytrauma). Since systemic injuries and hemorrhagic shock affect mean arterial pressure, including polytrauma patients without statistical adjustment likely introduces variability and error into PRx calculations. Furthermore, specific included studies reported baseline clinical severity using broad categorical ranges (e.g., GCS ≤ 8) rather than providing precise median or mean admission GCS scores, which complicates direct comparisons of patient acuity across the reviewed literature.

Fourth, unmeasured physiological and pharmacological confounders affect the analysis. Cerebrovascular reactivity is sensitive to clinical interventions such as sedation depth, targeted temperature management, and PaCO2 variations. Most of the included retrospective models did not adjust for these variables, which might lead to an overestimation of the independent predictive value of PRx-derived metrics.

Fifth, calculating PRx requires continuous, artifact-free waveform data. While research settings use retrospective data-cleaning methods, real-time bedside monitors are susceptible to artifacts from line flushing, patient repositioning, or transducer miscalibration, which could potentially generate misleading prognostic targets in everyday clinical practice.

Finally, it must be acknowledged that the current evidence base for PRx is highly intellectually concentrated. A substantial proportion of the foundational research, dataset generation, and methodological development originates from a single academic society, specifically the neurotrauma research consortium at the University of Cambridge led by Czosnyka et al. [8,11,12]. While their contributions to the field are seminal, this concentration remains a limitation, as it underscores the need for broader external validation by independent research groups across diverse clinical environments.

## 5. Conclusions

In conclusion, current evidence suggests an associative relationship, indicating that PRx-derived metrics may offer additive prognostic value over standard ICP monitoring alone for mortality and functional outcome in severe TBI. However, these findings are derived from strictly observational data. Furthermore, alongside substantial methodological heterogeneity, the current literature is likely confounded by the use of continuous PRx monitoring predominantly in high-resource, specialist neurocritical care centers, a factor that could independently contribute to better patient outcomes. Therefore, while dynamic targets demonstrate strong physiological rationale, these institutional confounders currently preclude definitive clinical recommendations. Establishing the true clinical utility of PRx monitoring requires large, standardized, prospective multicenter trials to isolate its prognostic and therapeutic effects from institutional baseline care.

## Figures and Tables

**Table 1:** **Overview of included studies**.

Author (Year)	Study Design	Data Source and Sample Size (*n*)	Median Age and Admission GCS	Main Monitoring Metrics	Key Statistical Findings (PRx vs. ICP)	QUIPS Overall
Kim et al. (2019) [23]	Retrospective	Cambridge, UK, (*n* = 295)	Age: 36 GCS: 6	ICP, PRx	Mortality: Mean ICP and mean PRx were not statistically significant predictors (*p* > 0.05). However, cumulative duration of impaired autoregulation (PRx > 0.20) was significantly associated with mortality (AUC 0.64, *p* < 0.001). Incremental Value: In patients with intracranial hypertension (>22 mmHg), mean ICP yielded an AUC of 0.66. The duration of PRx > 0.20 showed higher discrimination (AUC 0.77), representing an AUC increase of +0.11 compared to ICP alone.	Low
Zeiler et al. (2020a) [24]	Prospective	CENTER-TBI, Europe, (*n* = 193)	Age: 51, GCS: 6	ICP, PRx	Incremental Value: Adding %Time PRx > 0.25 to the IMPACT Core + Mean ICP model increased AUC from 0.780 to 0.819 (*p* < 0.0001). The model explained an additional 10.4% of outcome variance (ΔPseudo-R^2^). Functional Outcome (GOSE): Adding PRx-derived metrics to baseline models did not significantly improve discrimination (*p* > 0.05).	Low
Zeiler et al. (2020b) [25]	Prospective	CENTER-TBI, Europe, (*n* = 43)	Age: 46, GCS: NR	ICP, PRx	Mortality: PRx > 0.25 was a stronger predictor of death (AUC 0.747, *p* = 0.002) compared to ICP > 20 mmHg (AUC 0.648, *p* = 0.006). Functional Outcome (GOSE): PRx > 0.25 was the only significant univariate physiological predictor (AUC 0.679, *p* = 0.034), whereas ICP > 20 mmHg was not statistically significant (*p* > 0.05).	Moderate
Stein et al. (2025a) [26]	Retrospective	Winnipeg, Canada, (*n* = 124)	Age: 42, GCS: 6.5	ICP, iICP	Mortality: PRx-based (iICP) predicted mortality better (AUC 0.660, *p* = 0.029) than standard ICP > 20 mmHg (AUC 0.614, *p* = 0.021). Incremental Value: Adding PRx-derived (iICP) into the IMPACT Core model increased AUC from 0.793 to 0.914 and provided greater additional variance (ΔR^2^ = 0.263) compared to standard ICP (AUC 0.855, ΔR^2^ = 0.156).	Low
Adams et al. (2017) [27]	Retrospective	Cambridge, UK, (*n* = 601)	Age: 39, GCS: NR	ICP, PRx	Mortality: The PRx-based model predicted fatal outcome best in the early phase (0–48 h: AUC 0.86) compared to the ICP-based model (AUC 0.80). The predictive power of the PRx model significantly decreased over time (AUC dropped to 0.74 at 240 h, *p* < 0.05). Incremental Value: Adding PRx to the static clinical model (baseline AUC approx. 0.69) significantly improved prediction accuracy in the first 48 h (AUC increased to 0.86). In the model, PRx remained an independent predictor (OR 11.43, *p* = 0.001) alongside ICP.	Low
Petkus et al. (2020) [28]	Retrospective	Vilnius, Lithuania (*n* = 81)	Age: 40, GCS: 5	ICP, LCAI	Mortality: Younger patients (<45 y) tolerated higher failure (LCAI > 100 min, *p* = 0.043; mean PRx > 0.36). Elderly patients (>45 y) had significantly lower tolerance: mortality occurred at LCAI > 61 min (*p* = 0.0196) and mean PRx > 0.26. Incremental Value: LCAI was a strong independent predictor of outcome (OR 29.62; 95% CI 2.09–419.07, *p* = 0.012) in a multi-factorial model achieving 88.6% accuracy. Standard ICP/CPP parameters did not improve this model’s accuracy further.	Moderate
Stein et al. (2025b) [29]	Retrospective	CAHR-TBI, Canada (*n* = 365)	Age: 38, GCS: 6	ICP, PRx, iICP	Mortality & Functional Outcome: Optimization analysis identified PRx > +0.05 as the superior threshold for deriving individualized ICP targets (iICP). This specific iICP yielded the highest discrimination for both mortality (χ^2^ = 5.48) and favorable outcome (χ^2^ = 7.79). Incremental Value: The PRx-derived iICP target offered superior physiological sensitivity, demonstrating a threefold stronger correlation with autoregulatory failure burden (r = 0.49) compared to the standard ICP > 20 mmHg limit (r = 0.14).	Low
Beqiri et al. (2023) [30]	Retrospective	CENTER-TBI, Europe, (*n* = 171)	Age: 53, GCS: NR	ICP, PRx, LLR	Mortality: Non-survivors spent significantly more time with CPP < LLR (Lower Limit of Reactivity) (*p* < 0.001, AUC 0.73). High ICP (>20 mmHg) was most lethal when coincident with CPP < LLR. Incremental Value: Adding PRx-based LLR to the IMPACT Core model significantly improved predictive performance (AUC 0.84 to 0.88; Adj. R^2^ 0.49 to 0.57; *p* < 0.001), outperforming models based on ICP alone.	Low
Eide et al. (2007) [31]	Retrospective	Cambridge, UK, (*n* = 76)	Age: 34, GCS: 7	ICP, PRx	Functional Outcome: PRx levels successfully discriminated between patient groups, being significantly higher in unfavorable outcome (0.20) vs. favorable (0.03, *p* < 0.01). Mean ICP also showed significant discrimination (22.8 vs. 16.2 mmHg, *p* < 0.01). Incremental Value: Multiple regression analysis confirmed that PRx acts as a significant independent predictor of outcome (*p* = 0.03) alongside Mean ICP (*p* = 0.04), indicating additive prognostic utility beyond pressure monitoring alone	Moderate

Abbreviations: CPP: Cerebral Perfusion Pressure; ICP: Intracranial Pressure; PRx: Pressure Reactivity Index; iICP: Individualized ICP threshold derived from PRx optimization; LCAI: Longest Continuous Duration of Autoregulatory Impairment; LLR: Lower Limit of Reactivity; GOS/GOSE: Glasgow Outcome Scale/Extended; AUC: Area Under the Receiver Operating Characteristic Curve; IMPACT: International Mission for Prognosis and Analysis of Clinical Trials in TBI. Definitions of PRx-Derived Metrics: 1. iICP (Individualized ICP Target): A dynamic, patient-specific threshold derived by plotting PRx against ICP to pinpoint the pressure level where cerebral autoregulation consistently fails. 2. LCAI (Longest Continuous Duration of Autoregulatory Impairment): A metric quantifying the sustained burden of vascular instability by measuring the longest uninterrupted duration of impaired reactivity. 3. LLR (Lower Limit of Reactivity): The lower limit of the autoregulatory curve, representing the specific CPP breakpoint below which vessels lose reactivity and become passive.

## Data Availability

The dataset was derived from previously published, publicly available literature.

## Supplementary Materials

The following supporting information can be downloaded at: https://www.mdpi.com/article/10.3390/jcm15124611/s1, File S1: PRISMA checklist; File S2: Database string; File S3: GRADE assessment.

## Abbreviations

The following abbreviations are used in this manuscript:

ABP	Arterial Blood Pressure
CPPopt	Optimal Cerebral Perfusion Pressure
LLR	Lower Limit of Reactivity
iICP	Individualized ICP threshold
PRx	Pressure Reactivity Index
GOS	Glasgow Outcome Scale
CPP	Cerebral Perfusion Pressure
LCAI	Longest Continuous Duration of Autoregulatory Impairment
MAP	Mean Arterial Pressure
TBI	Traumatic Brain Injury
AUC	Area Under the Receiver Operating Characteristic Curve
GCS	Glasgow Coma Scale
ICP	Intracranial Pressure
IMPACT	International Mission for Prognosis and Analysis of Clinical Trials in TBI
