# Supplementary material for: Prognostic Value of Cerebrovascular Reactivity (PRx) Versus Intracranial Pressure (ICP) Monitoring in Traumatic Brain Injury: Systematic Review
Source: J Clin Med. 2026 Jun 14;15(12):4611. doi: 10.3390/jcm15124611 (PMC13300955; doi:10.3390/jcm15124611)
Supplement: Supplementary file 1 [file jcm-15-04611-s001.zip › File S2.pdf]

PubMed:
("Craniocerebral Trauma"[Mesh] OR "Traumatic Brain Injury"[Title/Abstract] OR TBI[Title/Abstract] OR "severe head injury"[Title/Abstract] OR "acute brain injury"[Title/Abstract] OR "brain trauma"[Title/Abstract] OR "head trauma"[Title/Abstract] OR "closed head injury"[Title/Abstract])
AND
("Cerebrovascular Circulation"[Mesh] OR "pressure reactivity index"[Title/Abstract] OR PRx[Title/Abstract] OR "cerebral autoregulation"[Title/Abstract] OR "cerebrovascular reactivity"[Title/Abstract] OR "pressure-reactivity"[Title/Abstract] OR "autoregulatory reserve"[Title/Abstract])
AND
("Intracranial Hypertension"[Mesh] OR "Intracranial Pressure"[Mesh] OR "intracranial pressure"[Title/Abstract] OR ICP[Title/Abstract] OR "intracranial hypertension"[Title/Abstract] OR "brain swelling"[Title/Abstract] OR "raised ICP"[Title/Abstract])
AND
("outcome"[Title/Abstract] OR "prognosis"[Title/Abstract] OR "mortality"[Title/Abstract] OR "monitoring"[Title/Abstract] OR "neurological outcome"[Title/Abstract])
AND
(("2000/01/01"[PDAT] : "3000/12/31"[PDAT]) AND English[lang] AND Humans[Mesh] NOT (Review[pt] OR Meta-Analysis[pt] OR Editorial[pt] OR Comment[pt] OR Case Reports[pt]))

Scopus:
TITLE-ABS-KEY("Traumatic Brain Injury" OR TBI OR "severe head injury" OR "acute brain injury" OR "brain trauma" OR "head trauma" OR "closed head injury")
AND
TITLE-ABS-KEY("pressure reactivity index" OR PRx OR "cerebral autoregulation" OR "cerebrovascular reactivity" OR "pressure-reactivity" OR "autoregulatory reserve")
AND
TITLE-ABS-KEY("intracranial pressure" OR ICP OR "intracranial hypertension" OR "brain swelling" OR "raised ICP")
AND
TITLE-ABS-KEY(patients OR clinical OR outcome OR prognosis OR mortality OR monitoring OR "neurological outcome")
AND
PUBYEAR > 1999 AND LANGUAGE(english) AND DOCTYPE(ar)

WOS:
TS=("Traumatic Brain Injury" OR "TBI" OR "severe head injury" OR "acute brain injury" OR "brain trauma" OR "head trauma" OR "closed head injury")
AND
TS=("pressure reactivity index" OR "PRx" OR "cerebral autoregulation" OR "cerebrovascular reactivity" OR "pressure-reactivity" OR "autoregulatory reserve")
AND
TS=("intracranial pressure" OR "ICP" OR "intracranial hypertension" OR "brain swelling" OR "raised ICP")
AND
TS=(patients OR clinical OR outcome OR prognosis OR mortality OR monitoring OR "neurological outcome")

AND
LA=(English) AND PY=(2000-2030)
NOT DT=("Review" OR "Review Article" OR "Meeting Abstract" OR "Meeting" OR "Book" OR "Book Chapter" OR "Editorial Material" OR "Letter" OR "Case Report" OR "Data Set" OR "Dissertation Thesis" OR "Awarded Grant" OR "Early Access" OR "Abstract" OR "Data Study" OR "Retracted Publication" OR "Unspecified" OR "Patent")

PUBMED records:540
WebOfScience records: 388
Scopus records:373

Unique records: 766
Duplicates removed: 535
Total records identified: 1301
