# Supplementary material for: Prognostic Value of Cerebrovascular Reactivity (PRx) Versus Intracranial Pressure (ICP) Monitoring in Traumatic Brain Injury: Systematic Review
Source: J Clin Med. 2026 Jun 14;15(12):4611. doi: 10.3390/jcm15124611 (PMC13300955; doi:10.3390/jcm15124611)
Supplement: Supplementary file 1 [file jcm-15-04611-s001.zip › File S3.pdf]

GRADE Summary of Findings

Outcome (Endpoint)	No. of Studies (Design)	Risk of Bias	Inconsistency	Indirectness	Imprecision	Other Considerations	Certainty of Evidence (GRADE)
**1. Mortality Prediction**	**7** (Observational)	**Not serious**	**Serious** [a]	**Not serious**	**Not serious**	**None**	⊕⊕○○ **LOW**
**2. Incremental Prognostic Value**	**6** (Observational)	**Not serious**	**Serious** [b]	**Not serious**	**Not serious**	**None**	⊕⊕○○ **LOW**
**3. Functional Outcome (GOS/GOSE)**	**4** (Observational)	**Not serious**	**Very serious** [c]	**Not serious**	**Not serious**	**None**	⊕○○○ **VERY LOW**

[a] PRx threshold definitions varied across studies (PRx > 0.20, > 0.25, > 0.36), precluding direct comparability of AUC values.

[b] Heterogeneity in baseline models (IMPACT Core, static clinical), PRx metric type (iICP, %Time PRx, LLR, LCAI), and reported effect sizes (ΔAUC range: +0.039 to +0.121).

[c] Zeiler et al. (2020a) — the sole prospective study (n = 193) — found no statistically significant improvement in GOSE discrimination after PRx addition (p > 0.05); retrospective cohorts reported significant improvements in the same outcome.

Abbreviations: AUC = Area Under the ROC Curve; GOS/GOSE = Glasgow Outcome Scale (Extended); IMPACT = International Mission for Prognosis and Analysis of Clinical Trials in TBI; PRx = Pressure Reactivity Index; ⊕ = high-certainty component; ○ = low-certainty component.
